# Identifying Social Media‐Based Interactions That Help Adults to Adhere to Weight Loss Goals: A Systematic Review

**DOI:** 10.1111/obr.70030

**Published:** 2025-09-30

**Authors:** Constanze Betz, Mirna Al Masri, Laura M. König, Tina Bartelmeß

**Affiliations:** ^1^ Faculty of Life Sciences: Food, Nutrition, and Health University of Bayreuth Kulmbach Germany; ^2^ Faculty of Law, Business, and Economics University of Bayreuth Bayreuth Germany; ^3^ Faculty of Psychology University of Vienna Vienna Austria

**Keywords:** interactions, social inequality, social media, social support, weight loss

## Abstract

**Background:**

Social media may support weight loss through online interaction and support, but its impact on interactions, social support, psychological factors, and weight loss outcomes across socioeconomic groups is unclear. This review aimed to (1) identify social support mechanisms aiding weight loss on social media, (2) pinpoint effective platforms and functions, and (3) assess intervention effectiveness across diverse demographics.

**Methods:**

A comprehensive search of PubMed, PsycINFO, and Web of Science was conducted through mid‐2023. Studies included targeted adults without psychiatric disorders and linked social media use to outcomes like weight, diet, physical activity, self‐management, or social support. Studies not meeting these criteria were excluded.

**Results:**

From 61 studies, informational support was most common (83%), followed by esteem (52%), network (47%), and emotional support (44%). Tangible support was rare due to the need for physical proximity. Informational and esteem support showed positive effects, but challenges like social comparison and negative group dynamics were noted. Facebook was the most studied platform, with higher engagement linked to better outcomes. Factors such as network embeddedness, tailored support, and platform familiarity influenced effectiveness. Only 18 studies addressed social inequality, showing younger individuals and women benefit more from these interventions.

**Conclusions:**

Social media facilitates weight loss through diverse support mechanisms, but challenges like varied platform preferences and social inequality require attention. Tailored interventions and strategies to promote engagement and mitigate negative dynamics are critical for maximizing outcomes.

## 1 Background

Obesity and overweight are pervasive public health issues in high‐income countries, imposing significant economic burdens and elevating the risk of chronic diseases like cancer, cardiovascular disorders, and diabetes [1]. Emerging evidence suggests that harnessing social media platforms, such as Facebook or Twitter, offers a promising avenue for implementing behavioral weight loss interventions at the population level [2, 3, 4]. As of 2022, the global user base for social media surpassed 4.59 billion individuals [5], underscoring the imperative to develop tailored strategies specifically for individuals with overweight or obesity, as well as those seeking to lose weight, who frequently engage with these platforms. Prevalence rates of overweight and obesity as high as 75% have been reported among users of online weight‐loss communities in previous studies [6]. Social media, defined as “Internet‐based platforms that enable users to opportunistically engage in interactions and selectively self‐present, either synchronously or asynchronously, to both broad and targeted audiences who derive value from user‐generated content and the perceived social interaction” [7], facilitates participatory internet activities, expanding the dissemination and consumption of health‐related content [8]. Users actively share health experiences and opinions, fostering complex interactions on weight loss behaviors and goals [9]. The integration of social media into weight loss interventions confers distinct advantages over conventional in‐person approaches, particularly in enhancing social interactions and support, especially in societies where self‐monitored online weight‐loss communities have gained prevalence [10, 11]. Secondary analyses indicate that social media facilitates peer‐to‐peer social support, with users providing time, attention, and understanding, often grounded in shared living conditions or socioeconomic status (SES) [12, 13]. These analyses revealed several key findings: (1) group‐based interventions are more effective in achieving clinically significant weight loss compared to individual interventions, (2) peer support may be especially valuable in resource‐limited settings, and (3) nevertheless, group‐based interventions show attrition rates comparable to those of individual approaches. Drawing on the Social Support Behavior Code (SSBC) model [14], social support on social media can be classified into five core types (and 23 subtypes). These mechanisms, namely informational, tangible, esteem, network, and emotional support, play crucial roles in promoting and facilitating healthier behaviors and outcomes.

Social support significantly influences the modification of disease risk factors and behaviors in daily life, encompassing objectives such as weight loss, particularly in conditions like overweight and obesity [15]. Effective pursuit of weight loss goals necessitates social support and informed decision‐making regarding dietary and exercise behaviors [16]. Preliminary evidence from empirical studies and recent systematic reviews suggests that interventions integrating social media can effectively promote weight loss and enhance social interaction and support among various heterogeneous participants [17, 18, 19, 20]. However, the existing evidence in the field of weight loss interventions involving social media, and social media research in the context of weight loss more generally, remains fragmented, with uncertainties regarding the specific features of each social media platform and the mechanisms by which they influence participant interactions, social support, and subsequent weight loss behaviors and outcomes, particularly in different socioeconomic backgrounds [21].

Especially within high‐income countries, obesity prevalence is disproportionately higher among individuals with low SES [22]. At the same time, evidence is accumulating that behavioral interventions for promoting healthier lifestyles and weight management could be less effective in low‐SES individuals, at least under certain circumstances [23]. These disparities also extend to digital interventions [24, 25], indicating that low‐SES individuals tend to engage less with and benefit less from interventions than their high‐SES counterparts. Szinay et al. [24] suggest that social interactions might be an important factor in sustaining engagement with digital interventions, given that face‐to‐face interventions tend to produce greater effects than digital‐only interventions [24, 26]. Therefore, social media, by enabling connectivity and interaction among users, may serve as a critical mechanism to enhance intervention effectiveness through increased engagement, particularly within low SES populations that typically exhibit lower participation rates in (digital) interventions. At the same time, social status is associated with patterns of social media use [27]; positive and negative effects of including social media in weight loss interventions should thus also be carefully evaluated to avoid reinforcing health inequalities [28].

We, therefore, conducted a systematic review to examine the role of social media–facilitated social support in individual weight loss attempts in everyday life and behavioral weight loss interventions and their relationship to weight loss outcomes. Our central research questions seek (1) to elucidate the social support mechanisms through which social media interactions aid individuals in pursuing weight loss goals, (2) to identify social media platforms and platform‐specific functions conducive to social support provision and weight loss attempts, and (3) to assess intervention uptake (i.e., downloading or installing an intervention or joining a group), engagement (i.e., usage frequency, duration, subjective interest, or influence), and effectiveness (i.e., whether the intervention achieved the desired changes in a weight‐related outcome) across socio‐demographic characteristics associated with social inequality.

## 2 Methods

This systematic review follows the PRISMA 2020 checklist. The review protocol was registered on the Open Science Framework (OSF) on December 6, 2022 (https://doi.org/10.17605/OSF.IO/7ACM9).

### 2.1 Eligibility Criteria

In this systematic review, eligibility criteria, outlined in Table S1 in the supplementary material, followed the PICOS (population, intervention, comparison, outcomes, and study design) framework. Included were studies investigating adult individuals without psychiatric disorders utilizing various social media platforms for user interaction. The broad inclusion of a heterogeneous population reflects real‐world user behavior patterns. Exclusion criteria encompassed individuals with mental health disorders, given the potential impact of their conditions on usage patterns and the risk of symptom exacerbation (e.g., in cases of eating disorders) that cannot be excluded. Study designs and comparators were not restricted. Primary outcomes included changes in weight, dietary habits, physical activity, perceived social support, feelings of belonging, and knowledge about nutrition. Eligible studies explored associations between platform usage and outcomes, published in English or German peer‐reviewed journals between January 2000 and November 2022.

### 2.2 Search Strategy

In November 2022, a comprehensive search across PubMed (including MEDLINE), PsycINFO, and Web of Science was conducted using keywords and Medical Subject Headings (MeSH) terms (see Table S2). The search strategy included alternative spellings and synonyms, covering literature from 2000 onwards. Forward and backward citation tracking was performed using Google Scholar, and reference lists of included studies were screened. Records were imported into Covidence (Covidence systematic review software, Veritas Health Innovation) for deduplication, and titles and abstracts were screened against eligibility criteria by two independent authors (C.B. and M.A.M.). Full texts of provisionally included studies were retrieved and assessed independently. Conflicts were resolved through discussion between screening authors, with unresolved conflicts adjudicated by all contributing authors.

### 2.3 Data Extraction

The final selected papers underwent independent data extraction by authors CB and MAM using MAXQDA 2023 (VERBI software). A coding scheme was deductively developed prior to extraction, covering participant details, concepts, contexts, methodologies, and key findings relevant to the research questions. The scheme allowed for flexibility and in‐progress modifications, accommodating additions such as codes for outcomes (“engagement” or “other outcomes”) and SES differentiations. Discrepancies between authors were resolved through discussion or adjudicated by an additional author.

The presentation of results adheres to the Preferred Reporting Items for Systematic Reviews and Meta‐Analyses (PRISMA) statement [29], with the study selection process depicted in a PRISMA flow chart (see supplementary material, Figure S1). Given the focus on delineating existing literature rather than critically appraising a cumulative body of research, a risk of bias appraisal was omitted. Indicators of social inequality identified in the reviewed studies were categorized and reported according to the PROGRESS‐Plus framework, which includes dimensions such as age, gender, education, race and ethnicity, income, geographic location, employment, and social status [30].

### 2.4 Data Analysis

In addressing Research Questions (1) and (2), our systematic review aimed to identify the specific social media platforms, features, and social support mechanisms aiding overweight individuals in their weight loss endeavors. Through a process of collating and synthesizing coded segments, we systematically examined platforms, subtypes of social support, and outcomes. This methodological approach facilitated the identification of the prevalent patterns of different social support types and the elucidation of the associated social media platforms and features and related effects on weight loss. Nonetheless, it is imperative to acknowledge the inherent challenges in establishing clear causal relationships between support subtypes, mechanisms, and outcomes. These challenges are rooted in ambiguities in the conceptualization of social support across included studies, as well as the lack of explicit differentiation among various support types and outcomes. Many studies encompassed multiple subtypes of social support and combinations of different social media platforms without explicitly mentioning this or differentiating between them in the presentation of results, further complicating interpretation. Additionally, the variability in measurement methodologies across studies, particularly those examining multiple variables concurrently, exacerbated these challenges.

Concerning Research Question (3), which explores potential variations in intervention effectiveness linked to socio‐demographic characteristics associated with social inequality, we opted against conducting a meta‐analysis due to the substantial heterogeneity observed among the included studies in terms of exposures, sample demographics, study methodologies, and outcome measures. Consequently, we provide an integrated narrative summary of the findings to furnish a comprehensive overview of the results. For this purpose, we categorized and reported social inequality indicators based on the PROGRESS‐Plus criteria [30], and we differentiated between intervention uptake, engagement, and effectiveness. Uptake was defined as the process of downloading and installing a mobile intervention device or enlisting in a required group or service [31]; engagement was measured by usage frequency and duration, as well as subjective interest and influence [32]; effectiveness was defined as the extent to which the intervention led to the desired outcome [33].

## 3 Results

### 3.1 Description of Studies

The final sample comprised 61 articles, including 38 quantitative studies, six secondary analyses, nine qualitative inquiries, and eight mixed‐methods studies. Publications spanned almost two decades from 2004 to 2022, with the majority from 2017 (nine articles). Targeted population groups varied widely, including gender‐specific studies [34, 35, 36], age‐restricted cohorts [37, 38], and sociocultural contexts like college or workplace settings [36, 39, 40, 41]. Some studies have been conducted with the aim of cultural tailoring of interventions [42, 43, 44, 45] or for tailoring interventions for special circumstances [46]. Additionally, there are studies focusing on adults in general or those without a detailed sociodemographic overview [11, 47, 48, 49, 50, 51].

Offline social support was incorporated in some studies with reciprocity in the online context [6, 49, 52] and without reciprocity in the online context [40]. Platform usage context and utilized platforms vary considerably across the studies examined. Generally, platforms are used for interventions for study purposes; however, 18 papers investigate the natural use of and exposure to social media in people with weight loss goals. For the purposes of this study, social media is defined in accordance with Carr and Hayes (2015) as Internet‐based platforms that enable users to opportunistically engage in interactions and selectively self‐present, either synchronously or asynchronously, to both broad and targeted audiences who derive value from user‐generated content and the perceived social interaction [7].

### 3.2 Social Support Mechanisms in Weight Loss via Social Media

To address Research Question (1) concerning social support mechanisms facilitated by interactions on social media platforms in everyday life and among participants engaged in weight loss interventions, we examined the five core types of social support as delineated by Cutrona and Suhr [14] for evidence from the reviewed studies. Informational support involves sharing facts, advice, and resources, while tangible support includes sharing digital tools, performing tasks, and offering help. Esteem support provides validation and recognition, while network support facilitates connections. Emotional support encompasses encouragement, empathy, and nurturing relationships [14]. The results, summarized in Table 1, depict the distribution of the five primary support types across the study sample.

**TABLE 1:** Distribution of social support core types.

	Informational	Tangible	Network	Esteem	Emotional	Total
Arigo (2015)	X					1
Arigo et al. (2015)	X		X	X	X	4
Athanasiadis et al. (2015)	X		X	X	X	4
Atwood et al. (2018)	X	X	X	X	X	5
Ballantine and Stephenson (2011)	X				X	2
Cavallo et al. (2014)	X					1
Chang et al. (2021)	X		X		X	3
Das and Faxvaag (2014)	X		X	X		3
LaPena and Quintanilla (2015)	X	X	X	X		3
He et al. (2017)	X					1
Hutchesson et al. (2013)	X					1
Hwang et al. (2010)	X	X	X	X		4
Hwang et al. (2014)	X			X		2
Laranjo et al. (2020)			X			1
Liu and Lachmann (2021)	X			X		2
Liu and Yin (2020)	X					1
Maglalang et al. (2017)	X		X	X		3
Merchant et al. (2017)	X					1
McVay et al. (2022)	X				X	2
Mo et al. (2019)			X	X		2
Pagoto et al. (2014)	X		X	X	X	4
Pappa et al. (2017)					X	1
Pullen et al. (2008)	X					1
Razak et al. (2020)	X	X		X		3
Reading et al. (2019)	X			X	X	3
Reynolds et al. (2018)	X	X		X		3
Richardson et al. (2010)	X					1
Stotz et al. (2011)	X	X	X	X	X	5
Taiminen et al. (2016)					X	1
Turner‐McGrievy and Tate (2011)			X			1
Turner‐McGrievy and Tate (2013)	X		X	X	X	4
Wang et al. (2015)	X		X	X	X	4
Wang and Willis (2018)	X			X	X	3
Watanabe‐Ito et al. (2020)	X		X	X	X	4
Xu and Cavallo (2021)			X			1
Zhang and Jemmott (2019)	X				X	2
Σ	30	6	17	19	16	
% of 36 studies reporting social support mechanisms	83.3	16.6	47.2	52.7	44.4	
% of total 61 reviewed studies	48.4	9.7	27.4	30.6	25.8	

Notably, only 36 out of 61 studies adequately elucidated the specific types of social support examined. Nineteen studies employed questionnaire‐based measures of perceived social support, which, while providing evidence of social support within the context, may not fully elucidate support mechanisms [35], for example, in [53, 54, 55]. Regarding specific support types, informational support was reported in 83.3% (30/36) of the studies, followed by esteem support (52.7%), emotional support (44.4%), and network support (47.2%). Tangible support, understandably, was less frequently reported (16.6% of studies) due to its reliance on physical proximity. Nonetheless, opportunities for demonstrating tangible support in an online setting do exist, such as indicating willingness to assist others.

#### 3.2.1 Informational Support

Informational support, that is, involving the exchange of factual information with minimal barriers to entry, was prevalent across the reviewed studies. However, the absence of informational support in some studies may be attributed to the lack of conversational content samples provided by scholars, potentially introducing bias. Different sources of informational support were identified, including peer‐to‐peer interactions [34, 56, 57] and conversations with professionals or moderators [57, 58]. The reviewed studies report that conversations with professionals or at least moderated conversations increase the likelihood of factually correct statements [57, 58] or foster interaction at all (e.g., responses to weekly prompts as in [56] or Cavallo et al. [59]). In some cases, the informational support was provided by the moderator in terms of delivering educational information [46, 48]. Other content shared included giving and reading self‐statements, for example, regarding own experiences and accomplishments, goals and goal‐setting [6, 34, 37, 49, 50, 56, 57]. Furthermore, questions and answers [57, 60], and links or information on various topics such as recipes or physical activity instructions were prevalent [6, 11, 34, 36, 38, 40, 45, 50, 52, 61, 62, 63]. Textual frames were the primary mode of conveying informational support, although some studies also utilized images [6, 36, 49, 52, 57, 63], primarily on platforms like Facebook, Instagram, forums, and other apps.

Informational support served as a resource for easily retrievable facts [57, 58] and facilitated self‐comparison [34, 37, 56, 60, 64, 65]. It also reframed personal experiences through narratives modeled by similar cohorts in online environments, providing valuable experiences for participants [6, 47, 58]. The non‐judgmental nature of informational content was highlighted [62, 66], along with its role in relieving self‐blame [66] and providing motivation [6, 34, 43, 45, 50, 56, 67]. Additionally, informational support promotes accountability [43, 63, 66], a sense of agency [52], perceived support [68], and the formation of (migratory) friendships or connections [6, 49, 52, 63]. However, negative effects such as social comparison leading to dropout or negative group dynamics were also noted in one case [64].

#### 3.2.2 Tangible Support

Tangible support in online contexts was identified in only six studies, with four involving partial online tangible support and three demonstrating willingness to assist others [46, 47, 52]. Reciprocity was observed in studies by Hwang et al. [6], LaPena and Quintanilla [49], Stotz et al. [52], and Razak et al. [63], where tangible support transitioned from online connections to offline interactions, such as organizing walking groups or exercising together. Additionally, Merchant et al. [40] and LaPena and Quintanilla [49] reported instances of tangible support within pre‐existing personal networks, including activities like meeting for walks or exercising together.

#### 3.2.3 Network Support

Network support frequently manifests through actions such as leveraging specific contacts [47, 64] or constructing offline networks [40, 49]. The presence of network support within groups [6, 47, 69] and the establishment of communicative connections (within assigned partnerships) [34, 36, 57, 60, 62, 69] within utilized platforms were frequently reported.

The reviewed studies further reported that participants often reorganize themselves based on interests or common starting points to target support toward individual needs [70]. Notably, individuals with physical limitations highlighted the potential for an enriched “social life” through online platforms [58]. Network support facilitates communication in various degrees of anonymity, accommodating participants' preferences. While some value the anonymity offered by social media platforms, others appreciate the opportunity to form connections and friendships with like‐minded peers. Connections established online sometimes transition to offline settings, a phenomenon known as “migratory friendships” [71], highlighting the importance of network support in facilitating emotional well‐being [52, 63]. Network support fosters feelings of belonging or recognition [34, 57], reduces barriers through decreased distance [64], provides motivation [43, 49, 58], and instills a sense of agency among participants [43]. However, in some cases, the network provided by the study design is not viewed as useful by the participants [72, 73], potentially due to fostering social comparison through plain data without relevant background or relations, or preferring communication with health professionals [64] over peer interaction.

#### 3.2.4 Emotional and Esteem Support

Distinguishing between emotional and esteem support in the reviewed studies posed challenges due to the lack of provided conversational content for evaluation. Emotional support, as defined by Cutrona and Suhr [14], encompasses encouragement, prayer, listening with empathy, sympathy, confidentiality, physical affection, and fostering close relationships. Esteem support involves compliments, validation, and relief from blame. Although there is some overlap, particularly in areas such as encouragement and compliments, the categories of social support were only explained in a few cases in the reviewed studies, so they are presented jointly in this section.

Despite cases of embarrassment and reluctance to share sensitive information like weight or physical activity‐related data online [36, 40], emotional support was evident, reaching the extent of friending others [66]. Many participants realized a sense of belonging or identification with the group's goals and activities [11, 47, 51, 57, 62, 64, 66, 70]. Online environments were described as emotional “safe spaces” to share topics without judgment [34, 52, 62, 66]. Additionally, encouragement was described as a form of emotional support [45].

Encouragement and complimenting each other's success were common manifestations of esteem support, linked to warm feelings, motivation, and decreased stress [6, 36, 37, 46, 49, 52, 57, 58, 62, 68, 69]. Complimenting each other's success [6, 11, 34, 47, 60, 70] was also linked to warm feelings and motivation [57, 58, 66, 70], a decrease in stress [52] as well as adherence to weight‐loss goals (defined as completion of the weight‐loss intervention) [6, 46]. Participants perceived standing together and belonging to a group [43, 49], which facilitated openly discussing feelings and sharing problems [6, 58, 66]. Offline esteem support through encouragement and reminders of friends was also observed [40]. Despite these positive aspects, some participants experienced ambivalence in online contexts, with comments eliciting both positive emotions as well as stress or sadness [63].

### 3.3 Social Media Platforms and Platform‐Specific Functions for Weight Loss

Research Question (2) aimed to identify social media platforms and platform‐specific functions conducive to social support provision and weight loss attempts. The reviewed studies revealed a wide array of platforms, with authors utilizing a total of 31 different combinations. This heterogeneity complicates the provision of a comprehensive overview, as each platform incorporated some, but not necessarily all, characteristic features of typical social media—such as messaging, commenting, liking, group formation, befriending, and following. Communication within these platforms was predominantly peer‐to‐peer [47, 57, 74, 75], although in some instances, study team members facilitated or prompted interactions [39, 58, 76].

#### 3.3.1 Social Media Platforms

Twelve articles featured dedicated study websites, while Facebook (groups) is utilized in 13 studies. Eight studies incorporate Fitbit devices, either independently or in conjunction with online platforms. Some investigations focus solely on single platform elements, as exemplified by Pappa et al. [51] and Liu and Yin [50], who explore Reddit. Other studies employ study‐specific websites [38, 55, 77] or custom smartphone applications (apps) [42, 45, 72]. Community‐related apps like Noom or Asken are also prevalent [36, 40, 60, 73, 74]. Additionally, four studies explore WeChat's potential in weight loss interventions [37, 48, 64, 69]. Forums are primarily examined through qualitative [47, 58] or quantitative research [78], with commercial platforms being frequently encountered [6, 11, 55, 65, 66, 68, 79, 80, 81]. Other platforms, such as company intranets [82], MyFitnessPal [55], and Instagram [63], are explored to a lesser extent.

#### 3.3.2 Platform‐Specific Functions Used in the Weight Loss Context

Platforms containing self‐monitoring features, including calorie and physical activity tracking, were prevalent among intervention websites. While self‐monitoring has shown effectiveness [79], perceptions of its usefulness varied among participants, particularly between overweight and normal‐weight individuals. While overweight participants generally found tracking to be a helpful method to keep control, this was not the case for normal‐weight individuals [72]. With regard to social interactions, some platforms or study‐related websites offered possibilities to interact with shared self‐monitoring information, either with comment and like functions [34, 35] or via a diary version of self‐monitoring [36, 79].

Forums, also called discussion board activities, were influential in providing informational and emotional support, with higher engagement associated with better weight loss outcomes [78]. Use of discussion features in general is mentioned as providers of support and empathy [74, 83] and higher weight loss outcomes [79, 84] or better weight maintenance outcomes [85].

Concerning online connections among people already known to each other, some studies indicated no discernible benefits of community functions among people already familiar [56, 80]. Some studies point to privacy concerns when sharing sensitive health‐related data with known contacts on platforms such as Facebook [40]. Twitter, with its more anonymous structure, has been preferred in some cases [62], although its helpfulness varied [73]. However, the concept of familiarity remains complex. While too much closeness through real contact can be detrimental, strangers do not always offer easier access and connection either, as inhibitions need to be overcome [53, 63]. Being anonymous, on the other hand, can facilitate connections for some participants [72]. But the nature of online support, whether positive or negative, depends on group dynamics, with data sharing having both encouraging and privacy‐related implications [11, 53, 76, 78]. Individuals may rely on the accountability provided by others [21, 40, 64] but may also experience social comparison [40], which can have both positive and negative effects on motivation [40, 64] and weight loss efforts due to social norms or pressure [40, 64, 72].

### 3.4 Engagement With and Effectiveness of Interventions

Overall, engagement with social media and support function usage varied [21, 48, 67, 76, 77, 82], with higher engagement often associated with better outcomes in terms of physical activity and weight loss [37, 41, 44, 51, 54, 60, 70, 74, 79, 85, 86, 87, 88]. The impact of social media‐based interactions on adherence to weight‐loss goals is evident, although establishing a definitive relationship between features and outcomes is challenging due to methodological variations and issues with causality among variables. For instance, while Watanabe‐Ito et al. [36] attribute changes in eating behavior more to the educational content of interventions, Merchant et al. [40] observe an impact on daily life management, with participants adopting additional self‐management tools. Wang et al. [70] highlight the importance of accountability and adherence throughout all stages of weight management, emphasizing the transition to a maintenance stage as crucial and potentially identity‐interfering.

Studies report that particularly Fitbit has been utilized frequently by intervention participants [34, 35, 39, 43, 45]. The literature highlights several features conducive to positive reinforcement, such as a non‐judgmental environment [66] and engagement linked to better adherence and outcomes. Having likeminded individuals with similar backgrounds fosters trustworthiness and support, aiding in reframing personal experiences [6, 40, 49, 58].

Providing varied support tailored to individual preferences and user typologies is essential [51, 58, 75]. Interindividual differences in usage patterns—such as passive consumption versus active participation—significantly influence the type of support received, with passive users deriving more informational support and active users benefiting more from emotional support. Additionally, the level of user activity and communication directly affects the perceived support experienced by individuals [75]. Facilitators and moderators offer reliable health information [58, 64, 74], while peers facilitate authentic interaction and group participation [64, 74]. Participant feedback underscores the need for more guidance from study teams [42].

Network embeddedness, defined as the degree to which an individual is integrated into their social environment, was significantly associated with weight loss success; however, the data did not allow for the identification of specific platform features contributing to this effect [74, 86]. However, network embeddedness emerges as the most statistically significant variable for weight loss [86], indicating the closeness of an individual within a network enabling access to support. Despite concerns such as privacy issues or personal mismatch with online support structures, Juszyk and Gillson [53] found that their intervention influenced the communication of targeted individuals with real‐life contacts, leading to more conversations about healthy eating.

However, lurking, passive consumption of content without interaction was common in several studies [38, 67, 72]. In some cases, participants did not use the provided platform functions, such as community boards [56] or Facebook [53], or the study results even showed no usefulness of these features [42].

Many articles lacked detailed descriptions of specific platform functionalities, making it challenging to draw explicit conclusions about their effects. Overall, the studies revealed that factors such as familiarity within the network [60, 83], comfort in sharing personal experiences, and shared goals were important for increasing adherence and achieving weight loss goals. Familiarity is also crucial for fostering social support [83] and is often developed over time [60].

Table 2 provides a comprehensive summary of the findings regarding platform specific functions conducive to social support provision and weight loss attempts.

**TABLE 2:** Summary of core findings for social media interactions and weight loss.

Aspect	Key findings
Platforms used	31 platform combinations identified across studies Most common: study‐specific websites (*n* = 12), Facebook (*n* = 13), Fitbit (*n* = 8) Others: Noom, WeChat, Asken, Instagram, MyFitnessPal, forums, intranets, custom apps
Functions and benefits	Forums and group walls offer informational and emotional support Social features (e.g., likes, comments and messaging) foster a sense of belonging Self‐monitoring tools (e.g., activity/calorie tracking) are especially effective for overweight users
Connection types	Peer‐to‐peer connections more effective than family/friends Privacy concerns frequent, especially on Facebook Anonymity can aid openness, though not universally helpful Excessive familiarity may hinder honest engagement
Engagement and effectiveness	Higher engagement linked to better weight loss and physical activity Fitbit use (often combined with other tools) boosts adherence via positive reinforcement Supportive, like‐minded communities build trust Moderators help in structured settings; less crucial in organic peer groups Network embeddedness strongly predicts success
Challenges and limitations	Passive use (“lurking”) can benefit some, but demotivate others Platform features not always fully utilized or effective Variability across studies limits comparability and causal conclusions

### 3.5 Intervention Effectiveness Across Socio‐Demographic Characteristics Associated With Social Inequality

Table 3 outlines the number of studies that found significant differences in the outcome based on the inequality indicator compared to the total number of studies that discussed uptake, engagement, and/or effectiveness. Overall, only 17 of the included 61 studies reported on any social inequality indicator. A detailed overview of findings related to intervention effectiveness but also social support, psychological outcomes, and weight loss—stratified by socioeconomic factors—is provided in Table S3. Table S3 synthesizes key themes across studies and highlights observed variations in engagement and effectiveness based on variables like age, gender, or education.

**TABLE 3:** Social inequality indicators studied in relation to uptake, engagement, and effectiveness.

Social inequality indicator	Uptake (*k* = 2)	Engagement (*k* = 15)	Effectiveness (*k* = 6)
Age	2/2	6/7	0/2
Gender	1/2	2/5	3/4
Education	1/2	2/2	
Race and ethnicity		1/3	0/1
Income			
Location	1/2		
Employment			
Social status	1/2		

*Note: k* denotes the number of studies identified for uptake, engagement, and effectiveness, respectively. Empty cells indicate that the inequality indicator was not studied in relation to the outcome.

#### 3.5.1 Uptake

Only two studies investigated uptake [48, 81], that is, differences in downloading and installing a mobile intervention. Neve [81] indicates that participants who subscribed to the 12‐week intervention period were of higher SES, lived mainly in bigger cities, and were younger, and He [48] reports that participants who joined the online social network intervention group were younger, female, and had university/college degrees or above.

#### 3.5.2 Engagement

Fifteen studies discussed engagement, that is, usage frequency, duration, subjective interest, or influence, by social inequality indicator [6, 21, 38, 43, 44, 48, 59, 60, 75, 78, 79, 81, 83, 85, 89]. Regarding age, one study reported no difference [75], while four studies reported that young participants were more engaged in digital intervention, as evidenced by more contribution and involvement in the social network [48, 59, 60, 81]. At the same time, Funk [85] reported that consistent users were older, Maglalang [43] mentioned that older participants favored using the Fitbit accelerometer, and Harvey‐Berino [89] established that younger participants did not complete all assessments in the intervention.

Gender was examined in five studies [60, 75, 78, 79, 85], with one reporting that gender was not a relevant factor in perceived social support [78]. One study noted that men were more engaged in terms of diet entries and website registration, while in terms of online support, more women posted messages on the forum in Johnson [79]. Funk [85] reported that consistent users were male.

Two studies observed no difference by race/ethnicity [59, 60], while Funk [85] reported that consistent users were other than African American. Two other studies mentioned that more consistent users and participants who completed all the intervention assessments had higher education [85, 89].

#### 3.5.3 Effectiveness

Furthermore, six studies discussed the effectiveness in relation to social inequality indicators, that is, whether there were differences in whether the desired outcome was achieved based on social inequality indicators [41, 44, 48, 60, 72, 79]. Out of these studies, three mentioned no difference by either age, gender, or race [41, 44, 79]. However, two studies showed that men lost more weight compared to women [48, 79], while in Laranjo [72], men increased their weight compared to women.

## 4 Discussion

Informational social support emerges as the most prevalent type of support among examined interventions, albeit with biases stemming from methodological disparities and targeted outcomes. Differences in needs and personal communication preferences also influence how social support is taken up. Among the studies, due to great heterogeneity, a best‐fit platform could not be identified, and fit largely depends on the targeted group as well as explicit aim. While only a minority of the studies investigated social inequality indicators, results suggest that especially age might be related to meaningful use of social media features. The heterogeneity of the study populations as well as study designs reflects the broader challenges associated with weight management in the general population but can contribute to a deeper understanding of the complex mechanisms and decision‐making processes that influence the effectiveness of social media‐based interventions. Differences in terminology and conceptualization of social support among reviewed works limit the possibilities for synthesis. Diverse study designs coupled with the wide array of social media platforms used complicate comparability. Nevertheless, core results can be summarized regarding social media‐based social support and promising social media platforms.

Participants' familiarity with technology and social environments significantly influences their comfort in seeking support within provided networks. Communication styles, platform preferences, and the presence of ‘lurkers’ affect both the perception and provision of emotional support. While Facebook's broad reach is favored, closed groups are preferred for privacy maintenance. Twitter (now X) is favored for weight loss discussions due to its less interconnected network and anonymity in comparison to Facebook [40].

However, closed Facebook groups may lack interactivity, highlighting the need for effective communication styles to enhance emotional support provision in weight loss interventions on social media. Passive and non‐reciprocal communication styles are observed among users [75], underscoring the phenomenon of lurking, which falls outside the construct of social support.

Different usage patterns among cohorts may explain disparate results. While in some cases, the cohorts have been higher grade users of the online social network or at least felt comfortable doing so, other cohorts barely used the target platforms, eventually out of discomfort with the platform, technology, unfamiliar people, or similar. Evolving familiarity through low‐barrier communication channels and privacy control enables access to social support. Upon achieving a sense of comfort to share, individuals may access more intimate forms of support, such as esteem and emotional support. Conversely, if familiarity is not established, support is likely to remain at superficial levels that necessitate minimal personal involvement. Yet, uncontrolled familiarity within networks may have detrimental effects [62], emphasizing the importance of creating safe [66], nonjudgmental spaces. Likewise, insights from studies by Kim et al. [74] and Xu and Cavallo [21] imply that achieving optimal support for individuals may not hinge on larger networks but rather on increased personal involvement to foster network embeddedness.

Support exchanged via digital platforms is predominantly informational in nature, which is consistent with the context of interactions often occurring among unfamiliar individuals. Informational support represents a low‐threshold form of engagement, requiring minimal relational investment. In contrast, the presence of emotional and tangible support within digital environments highlights the broader potential of these platforms to facilitate more meaningful forms of interaction. However, existing literature also underscores the associated risks. Social comparison processes—well documented in offline settings—are similarly activated in online contexts [40]. While such comparisons can serve as motivational drivers, particularly when individuals aspire to emulate the successes of others, they may also have adverse effects, including reduced self‐efficacy, demotivation, or, in extreme cases, the adoption of maladaptive health behaviors [64, 72]. Our findings suggest that rather than the specific social media platforms themselves, it is the presence and utilization of particular functional features that drive effective social support and positive behavior change in weight loss interventions. Interaction and support consequentially can also occur within platforms typically classified as digital health tools and similar, where they constitute core elements of digital behavior change tools in general [90]. This functional perspective highlights the importance of fostering interactive elements that create a sense of belonging and enable self‐monitoring, regardless of the platform used. Given the wide variety of platforms employed in the included studies, future research should focus on optimizing these core functions and understanding how they can be best tailored to individual user preferences to maximize engagement and intervention success.

As outlined in the results section and Table S3 in the supplementary material, differences in user engagement, social support, and intervention outcomes are evident across socioeconomic groups. Thus, media‐based weight loss interventions might not benefit all users equally, providing further support for a digital health divide. This became especially evident for engagement with, but also the effectiveness of, social media‐based weight loss interventions, which mirrors the results of an earlier systematic review on mobile interventions for weight‐related behaviors [24]. Inequalities mainly stemmed from age, with younger individuals being more involved in social media. This is in line with usage statistics that indicate that younger age groups are more frequent users of social media [91]. Importantly, age gaps may differ between social media platforms; however, due to the small number of studies investigating any social inequality indicator, we were unable to draw meaningful conclusions regarding the specifics of different platforms. Gender differences in engagement vary, with men more involved in some respects, such as self‐monitoring, while women engage more in online support forums. This is again in line with prior research that indicates that men engage more with core intervention features in weight loss interventions [92, 93]. At the same time, women are more likely to form connections on social networks [94], which suggests that women might benefit more from social support features in interventions than men [95]. Education also influences engagement, with higher‐educated individuals showing more consistent participation. This may point toward the crucial role of digital literacy for effective engagement with behavioral interventions also on social media [96, 97]. Regarding effectiveness, while some studies show no significant differences, others indicate gender‐based variations in weight loss outcomes. Although women are often more engaged with health‐related concerns and body image, recent evidence from an umbrella review suggests that men may derive greater benefit from digital health interventions. This disparity may be attributable to fewer competing demands, such as caregiving responsibilities, as well as physiological differences between sexes [98]. Notably, completion rates also differ by socioeconomic factors, with inconsistent findings across studies. Results on uptake were again sparse and mixed, highlighting the urgent need for more research, particularly in this area, given that intervention uptake is a prerequisite for effectiveness. In this context, greater consideration should be given to recruitment strategies, as they may systematically favor specific subpopulations—particularly those with higher digital literacy and greater trust in research and healthcare institutions [99].

Previous reviews on digital health intervention already indicated a limited scope where studies emphasize certain social indicators such as age, gender, and education, while neglecting other key factors [28]. This was reproduced in this systematic review where studies mainly focused on age, gender, and education. This lack of insight inhibits a comprehensive understanding of social inequality indicators and their impact on digital health interventions. This underscores the importance of broadening sample representation and incorporating social determinants throughout all phases of the research process to better understand the underlying factors contributing to the digital health divide [99]. Notably, one study highlights the potential of digital tools to empower populations with physical limitations, emphasizing the need for inclusive research approaches [58].

The results of the present systematic review underline that tailoring weight loss interventions to individual needs and preferences is crucial for their acceptance and effectiveness. Beyond the general recommendations to tailor goals and adapt content presentation to cultural preferences in digital interventions [100, 101], social media‐based interventions offer further opportunities for customization. This includes adjusting the type and frequency of social interactions [58] as well as tailoring discussion topics to align with individual participant preferences [51, 75]. Moreover, customization of communication styles, preferences for familiarity, platform selection, and other factors should be carefully aligned with the characteristics of the target population [43]. Thus, potential intervention users should be involved in the intervention development process [102]. Indeed, stakeholder involvement is related to improved clinical trial enrollment and retention [103], increased relevance [104], and potentially increased intervention engagement and effectiveness [105].

## 5 Limitations and Future Research

This study offers valuable insights but is subject to several limitations. First, the reliance on self‐reported data in many of the analyzed studies may introduce response bias, compromising reliability. Additionally, the heterogeneity of study designs, measurement methods, and populations limits direct comparisons and the generalizability of findings. The dynamic nature of online networks and the rapid evolution of technology further complicate efforts to comprehensively assess the interplay between social support and weight loss outcomes, especially over a 20‐year period. A notable limitation is that the literature search included studies across the entire population and weight spectrum, without a specific focus on populations with overweight or obesity, potentially overlooking differences in weight loss needs between individuals with obesity and those in the normal weight range. Another limitation of the study is the lack of clarity on whether the analyzed aspects of social media support and interaction provide greater benefits for weight loss compared to lower levels of social media engagement. Future research is needed to determine whether such an effect exists and, if so, to quantify its magnitude to evaluate its clinical relevance accurately. Furthermore, potential publication bias and language restrictions may have constrained the scope and representativeness of the included studies. Finally, inherent challenges in analyzing secondary data, such as methodological variability, uncontrolled confounding variables, and the inability to establish causality, limit the depth of interpretation, particularly regarding variations in reported social support or specific features of social media platforms.

Despite these limitations, this review makes a significant contribution by synthesizing current knowledge on the role of online social support in weight loss. It provides a comprehensive overview of the field, integrating findings from diverse studies to elucidate how online networks influence weight loss behaviors in everyday life among a heterogeneous population and in intervention contexts. The review also advances theoretical understanding by exploring the mechanisms through which online social networks affect weight loss. By identifying gaps and inconsistencies in existing literature, it highlights priorities for future research. Specifically, further studies are needed to distinguish platform‐specific characteristics, establish causal relationships in social support mechanisms, and confirm the hypothesis that informational support, while more accessible, is generally less effective than other types of support. Additionally, hypotheses regarding the role of network involvement in optimizing support provision warrant investigation. As different platform types facilitate varying modes of communication and users prefer distinct styles of engagement, designing effective social media‐based weight loss interventions remains a critical challenge for future research.

## 6 Conclusion

The impact of social support mechanisms is highly dependent on the preferences of individual users and is also linked to the type of medium used, as well as the perceived novelty and associated uncertainties of that medium. Further research with stringent methodology is needed to extract influences or causalities of individual platform characteristics on social support mechanisms. This also extends to the uptake of, engagement with, and effectiveness of social media‐based weight loss interventions in different subpopulations to ensure that existing health inequalities are not further exacerbated.

## Author Contributions

T.B. and L.M.K. were responsible for the overarching construction and management of the review. C.B. and M.A.M. led data extraction and analysis. T.B. supported data extraction and analysis. C.B. and T.B. were responsible for supporting the methodological approach to the review and the writing of the article. M.A.M. and L.M.K. provided critical edits to the manuscript. All authors read and approved the final manuscript.

## Ethics Statement

The authors have nothing to report.

## Consent

The authors have nothing to report.

## Conflicts of Interest

The authors declare no conflicts of interest.

## Supporting information


**Table S1:** PICOS statement and resulting eligibility criteria.
**Table S2:** Example Search String.
**Table S3:** Socioeconomic Differences in the Use and Impact of Social Media in Health Interventions.

**Figure S1:** PRISMA Flow Chart.

## Data Availability

All data generated or analyzed during this study are included in this published article (and its Supporting Information files).
