# Supplementary material for: Identifying Social Media‐Based Interactions That Help Adults to Adhere to Weight Loss Goals: A Systematic Review
Source: Obes Rev. 2025 Sep 30;27(3):e70030. doi: 10.1111/obr.70030 (PMC12926626; doi:10.1111/obr.70030)

Supplementary Information

Identifying social media-based interactions that help adults to adhere to weight loss goals: a systematic review

Constanze Betz*, Mirna Al Masri*,**, Laura M. König *,***, Tina Bartelmeß*

*Faculty of Life Sciences: Food, Nutrition, and Health, University of Bayreuth, Kulmbach, Germany

** Faculty of Law, Business, and Economics, University of Bayreuth, Germany

*** Faculty of Psychology, University of Vienna, Austria

Corresponding author: Tina Bartelmeß, tina.bartelmess@uni-bayreuth.de, Faculty of Life Sciences: Food, Nutrition, and Health, University of Bayreuth, Fritz-Hornschuch-Straße 13, 95326 Kulmbach, Germany

Table S1. PICOS statement and resulting eligibility criteria.

**Element**	**Definition**	**Inclusion criteria**	**Exclusion criteria**
Participants	Adults, i.e. individuals aged 18 years and older from the general population who do not suffer from psychiatric disorders including eating disorders	Adults in the general population	Children and adolescents under the age of 18<br><br>Populations with psychiatric disorders (e.g., eating disorders)
Intervention	Use of a social media platform (e.g., Facebook, TikTok, Twitter, Snapchat, Instagram, TripAdvisor) to promote interaction between users	Use of at least one of the following social media platforms to promote interaction between users: Instagram, TikTok, Reddit, Pinterest, Twitter, Facebook, WeChat, Snapchat, TripAdvisor, YouTube, Chefkoch, Recipe sharing sites, blogs, Discussion forums	Other digital interventions not using one of the listed social media platforms<br>Interaction through social media not incorporated<br>Studies focusing only on social usage frequency/ other parameters of platform use
Comparison	Any study design (experimental, quasi-experimental, or observational)	-	-
Outcomes	Weight, diet/ eating, or physical activity<br>Changes in perceived social support, social ties, belonging<br>Self-management, facilitation of implementation in everyday life<br>Social interaction<br>Changes in (perceived) knowledge and attitudes regarding nutrition(-al values)	Weight change, change in diet/eating or physical activity behavior, changes in perceived social support/ possibilities and participation, social ties, sense of belonging and social inclusion, Self-management, facilitation of implementation in everyday life<br>Social interaction<br>Changes in (perceived) knowledge and attitudes regarding nutrition(-al values)	
Study designs	Quantitative, qualitative or mixed methods studies including randomized-controlled trials, non-randomized studies, and observational studies	Quantitative, qualitative or mixed methods studies including randomized-controlled trials, non-randomized studies, and observational studies	

Additional criteria		Full texts available in English or German<br><br>Articles reporting on primary research<br><br>Articles published in peer-reviewed outlets (i.e., journal publications)	Full texts in any other language<br><br>Articles not containing primary data of individual studies (e.g., systematic or narrative reviews)<br><br>Articles published in outlets that do not provide peer-review (e.g., book chapters; theses; preprints)

Table S2: Example Search String

Database	Searchstring
WebofScience	(((TS=(Obesity OR overweight OR weight*)) AND TS=("social media" OR "social network" OR TikTok OR instagram OR facebook OR twitter OR reddit OR snapchat OR WeChat OR Pinterest OR web-based OR forum* OR "recipe shar* site*" OR youtube OR blog*)) NOT TS=(babies* OR baby* OR kid* OR child* OR children OR toddler* OR adolescen* OR infant* OR preschool OR schoolchild OR childhood OR newborn OR minors OR teen OR Teenage* OR teens OR boy OR boys OR girl OR girls)) AND (DT==("ARTICLE") AND LA==("ENGLISH" OR "GERMAN"))
PubMed	(((("Obesity"[Title/Abstract] OR "overweight"[Title/Abstract] OR "weight*"[Title/Abstract]) AND ("social media"[Title/Abstract] OR "social network"[Title/Abstract] OR "TikTok"[Title/Abstract] OR "Instagram"[Title/Abstract] OR "Facebook"[Title/Abstract] OR "twitter"[Title/Abstract] OR "reddit"[Title/Abstract] OR "snapchat"[Title/Abstract] OR "WeChat"[Title/Abstract] OR "Pinterest"[Title/Abstract] OR "web-based"[Title/Abstract] OR "blog*"[Title/Abstract] OR "forum"[Title/Abstract] OR "youtube"[Title/Abstract])) NOT ("babies*"[Title/Abstract] OR "baby*"[Title/Abstract] OR "kid"[Title/Abstract] OR "child*"[Title/Abstract] OR "children"[Title/Abstract] OR "toddler*"[Title/Abstract] OR "adolescen*"[Title/Abstract] OR "infant*"[Title/Abstract] OR "preschool"[Title/Abstract] OR "schoolchild"[Title/Abstract] OR "childhood"[Title/Abstract] OR "newborn"[Title/Abstract] OR "minors"[Title/Abstract] OR "teen"[Title/Abstract] OR "teenage*"[Title/Abstract] OR "teens"[Title/Abstract] OR "boy"[Title/Abstract] OR "boys"[Title/Abstract] OR "girl"[Title/Abstract] OR "girls"[Title/Abstract])) AND ("English"[Language] OR "German"[Language]) AND (("English"[Language] OR "German"[Language]) AND 2000/01/01:2022/12/31[Date - Publication])) AND (english[Filter] OR german[Filter])

Table S3: Socioeconomic Differences in the Use and Impact of Social Media in Health Interventions

**Domain**	**General Findings**	**Differences by Socioeconomic Group**
**Interaction (Engagement & Use)**	• Interaction patterns vary widely across studies.<br>• Passive use (e.g., lurking) is common.<br>• Closed Facebook groups popular but may lack interactivity.<br>• Twitter's anonymity increases user comfort for some.<br>• Participation influenced by personal comfort, platform fit, and digital familiarity.<br>• Preferences and engagement are highly individualized.	• **Age**: Younger users more active; older adults often disengaged.<br>• **Education**: Higher education correlates with better engagement, likely due to greater digital literacy.<br>• **Gender**: Men engage more with self-monitoring; women participate more in forums.<br>• **Digital Divide**: Lower SES groups and less tech-savvy users show reduced engagement.
**Social Support**	• Informational support is most common due to low emotional involvement.<br>• Emotional/esteem support typically accessed after building familiarity.<br>• Perceived support influenced by anonymity, platform type, and network size.<br>• Lurkers may gain less emotional benefit. - Platform customization improves support quality.	• **Gender**: Women more likely to seek and benefit from emotional support.<br>• **Education**: Higher-educated users more adept at navigating support systems.<br>• **Age/Digital Literacy**: Older and less tech-proficient users less likely to access emotional support.<br>• **Social Inequality**: Rarely examined explicitly, though evidence suggests disparities in access and benefit.
**Psychological Outcomes**	• Evidence is mixed due to heterogeneity in study designs.<br>• Social comparison can be either motivating or discouraging.<br>• Supportive, non-judgmental environments critical for positive outcomes.<br>• Emotional support may enhance well-being when accessed.<br>• Familiarity can sometimes lead to unwanted exposure and distress.	• **Limited Evidence**: Few consistent SES-based patterns.<br>• **Marginalized Groups**: May benefit less or face greater psychological risk due to discomfort or limited digital access.<br>• **Youth**: More familiar with digital norms, potentially experiencing greater psychological benefit.<br>• **Inclusive Design**: May help mitigate risks for vulnerable populations.
**Weight Loss Outcomes**	• Results are mixed; often measured indirectly.<br>• Informational and motivational support can enhance adherence.<br>• Engagement is a key mediator of success. - Personalized and interactive features improve outcomes.<br>• Larger networks do not guarantee better results – individual involvement is critical.	• **Gender**: Men may experience better outcomes, possibly due to fewer caregiving duties and physiological factors.<br>• **Education**: Higher education associated with greater engagement and better outcomes.<br>• **Digital Divide**: Reduced access and literacy in lower SES groups linked to poorer outcomes.<br>• **Uptake**: Disadvantaged groups show lower uptake and completion, highlighting the need for tailored recruitment and intervention strategies.

Figure S1: PRISMA Flow Chart

From: Page MJ, McKenzie JE, Bossuyt PM, Boutron I, Hoffmann TC, Mulrow CD, et al. The PRISMA 2020 statement: an updated guideline for reporting systematic reviews. BMJ 2021;372: n71. doi: 10.1136/bmj.n71

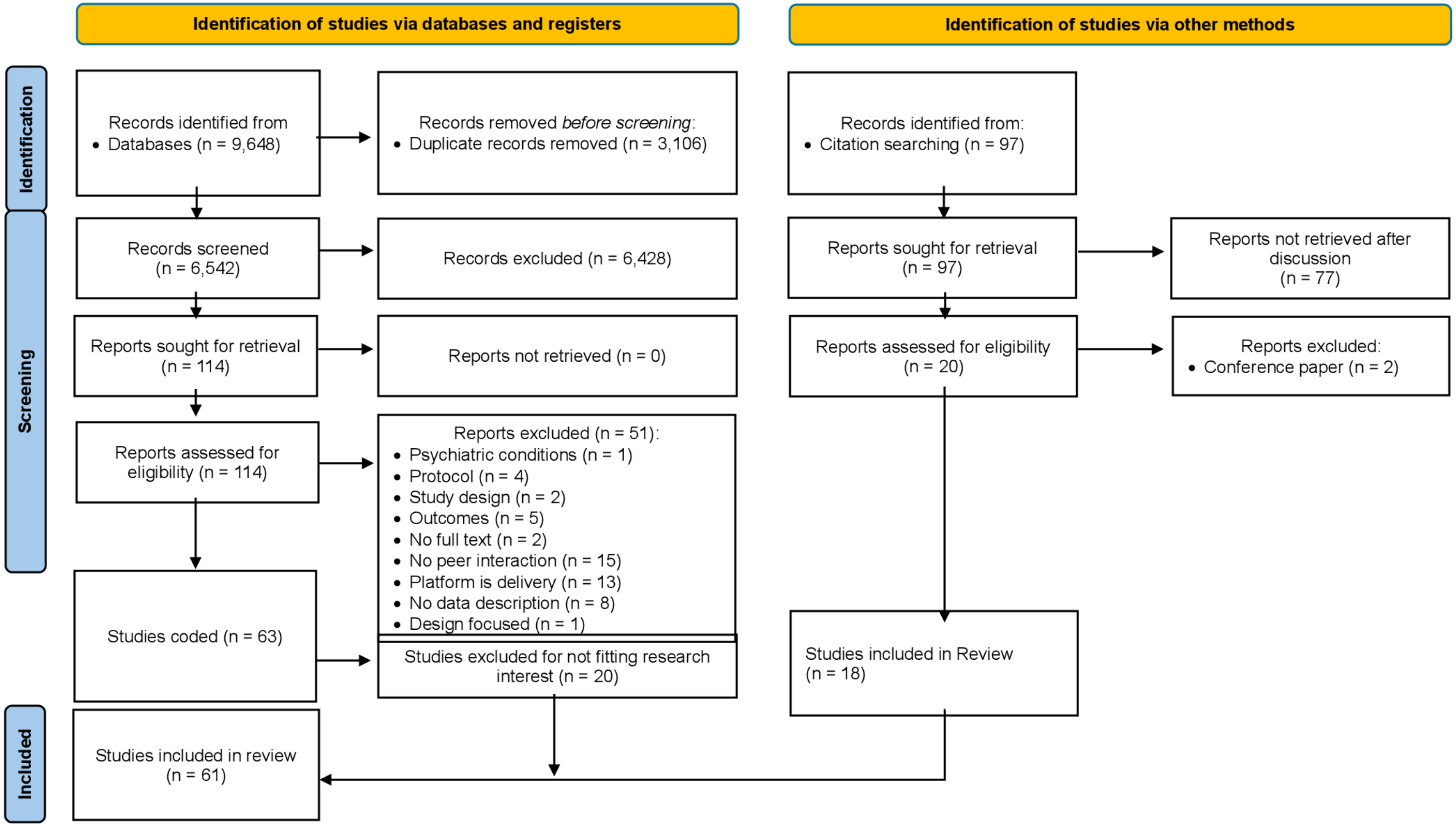

Supplement: Supplementary file 1 — Table S1: PICOS statement and resulting eligibility criteria. Table S2: Example Search String. Table S3: Socioeconomic Differences in the Use and Impact of Social Media in Health Interventions. [file OBR-27-e70030-s002.pdf]
