# Supplementary figures and images for: Identifying Social Media‐Based Interactions That Help Adults to Adhere to Weight Loss Goals: A Systematic Review
Source: Obes Rev. 2025 Sep 30;27(3):e70030. doi: 10.1111/obr.70030 (PMC12926626; doi:10.1111/obr.70030)

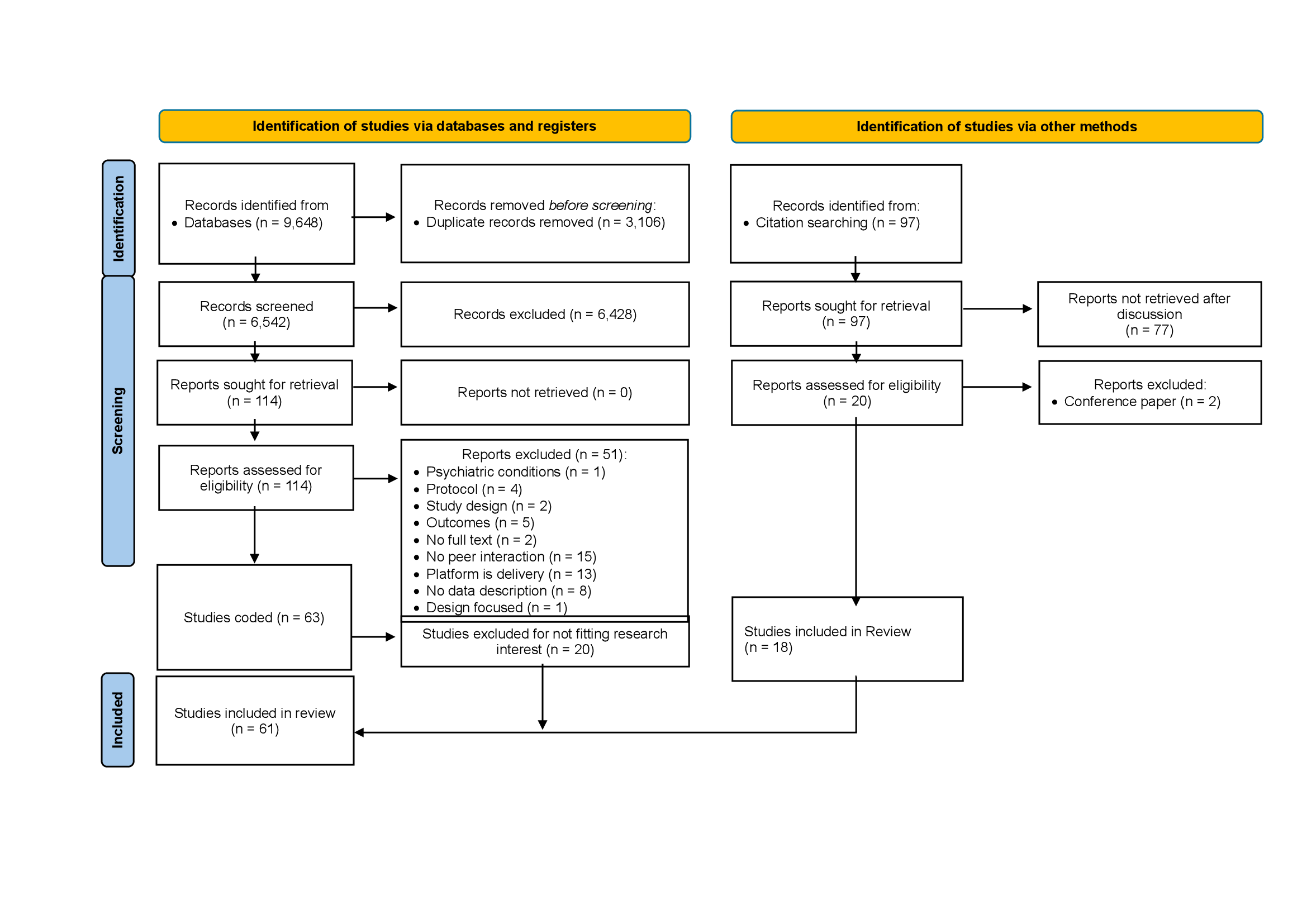

Supplement: Supplementary file 2 — Figure S1: PRISMA Flow Chart. [file OBR-27-e70030-s001.tiff]
